# The impact of age, performance status and comorbidities on nab-paclitaxel plus gemcitabine effectiveness in patients with metastatic pancreatic cancer

**DOI:** 10.1038/s41598-022-12214-4

**Published:** 2022-05-17

**Authors:** Martina Catalano, Giuseppe Aprile, Raffaele Conca, Roberto Petrioli, Monica Ramello, Giandomenico Roviello

**Affiliations:** 1 Department of Human Health Sciences, School of Human Health Sciences, University of Florence, Largo Brambilla 3, 50134 Florence, Italy; 2 Department of Oncology, San Bortolo General Hospital, AULSS8 Berica, Vicenza, Italy; 3 Division of Medical Oncology, Department of Onco-Hematology, IRCCS-CROB, Referral Cancer Center of Basilicata, via Padre Pio 1, 85028 Rionero, Vulture, PZ Italy; 4 Department of Medicine, Surgery and Neurosciences, Medical Oncology Unit, University of Siena, VialeBracci–Policlinico“LeScotte”, 53100 Siena, Italy; 5 Oncology Unit, Department of Medical, Surgical and Health Sciences, University of Trieste, Piazza Ospitale, Trieste, Italy; 6 Department of Health Sciences, University of Florence, viale Pieraccini, 6, 50139 Florence, Italy

**Keywords:** Biophysics, Cancer, Chemical biology

## Abstract

Few studies have evaluated the impact of risk factors such as performance status (PS) and comorbidities on overall survival (OS) in patients with metastatic pancreatic cancer (mPC). We investigated the influence of comorbidity, PS and age on nab-paclitaxel and gemcitabine (NabGem) effectiveness profile in naive patients with mPC. 153 patients with mPC treated with NabGem upfront was divided in three groups (score 0 to 3) based on the absence or the presence of one or more risk factors among: age ≥ 70 years, PS 1 and comorbidities and the clinical outcomes was compared. Fifty-five patients were elderly (≥ 70 years), 80 patients have PS 1, whereas the other have PS 0. Patients with no risk factors (score 0) had an overall survival higher (20 months) than patients with one or two risk factors (score 1–2) (OS 11 months) and with three risk factors (score 3) (OS 8 months) (*p* < 0.01). The difference in OS was also statistically significant in patients without comorbidities (OS 15 months) compared to those with ≥ 1 comorbidity (OS 10 months) (*p* < 0.001). NabGem chemotherapy represent an effective treatment in naive patients. Age, PS, and comorbidities were prognostic factors in patients with metastatic pancreatic cancer.

## Introduction

Pancreatic cancer (PC) is the fourth leading cause of cancer-related death in Europa and United States^1,2^. Most patients with PC are diagnosed with advanced stage, and the 5-year survival ranged from 5 to 10%^3^. With the aging of population, the number of elderly cancer patients has increased worldwide^4^. Pancreatic cancer often affects elderly patients with an average age at diagnosis of around 72 years^5^. An ever-increasing part of these elderly patients is affected by comorbidities and impaired organ function that often lead to inappropriately treatment in clinical practice based on the perception of reduced life expectancy and the ability to tolerate potential therapies side effects.

Therefore, sometimes less aggressive treatment options or best supportive care alone is offered to elderly patients due to the potential increase in toxicity. In systematic reviews, the use of chemotherapy has been shown to improve survival in advanced PC^6^. However, data on the benefits and toxicity of chemotherapy in elderly patients are very limited due to the low enrollment of this population in clinical trials^7^.

For more than a decade, gemcitabine has been the standard chemotherapy against advanced pancreatic cancer inasmuch it showed to prolong survival and improved clinical benefit response^8^. Two studies evaluated gemcitabine-containing regimens in elderly patients with pancreatic cancer, which however, have included only patients with good performance status (PS) and few comorbidities, rarely corresponding to patients in clinical practice^9,10^. Although with discordant results, gemcitabine-based treatment and dose-adapted fluorouracil combination regimens seem to be effective and well tolerated in these patients, and new combination regimens such as nab-paclitaxel and gemcitabine (NabGem) are evaluating^11^.

Chronological age alone, although associated with age-related impairment in organ function, does not well reflect the general physical status of patients. To date, is controversial which of the factors such as PS, comorbidity, age, have the most relevant impact on the treatment results in the elderly^7^. The interplay between age, comorbidity, and PS in predicting outcome in metastatic cancer is poorly understood. The Charlson Comorbidity Index (CCI) is the most widely used tool to measure comorbidity in patients with a prognostic implication in the adjuvant setting^8–11^. Therefore, an appropriate patient selection including multiple factors (*e.g.,* age, PS, comorbidities) and a proper balance of potential treatment benefits and side effects represent a crucial point for managing patients with PC to maximize the therapeutic benefit. The aim of this retrospective study was to clarify the impact of age, performance status and comorbidities on prognosis in patients with advanced pancreatic cancer.

## Results

### Patients’ characteristics

Between January 2015 and December 2018, 153 patients diagnosed with mPC and treated with NabGem have been identified. Baseline characteristics are shown in Table 1.

**Table 1:** Patient characteristics.

	All patients (N = 153)	Subgroup patients		
		Score = 0 (N = 28)	Score = 1–2 (N = 98)	Score = 3 (N = 27)
**Age, years**				
Median (range)	67 (50–84)	59.5 (49–69)	66 (50–83)	74 (70–84)
≥ 70	55 (35.9%)	0	28 (28.6%)	27 (100%)
**ECOG PS**				
1	80 (51.2%)	0	53 (54.1%)	27 (100%)
**Sex**				
Male	88 (57.5%)	14 (50%)	57 (58.2%)	17 (63%)
**N of comorbidities**				
≥ 1 comorbidities	95 (62.3%)	0	68 (69.4%)	27 (100%)
**CA 19.9—U/ml**				
Median Range	547 (0.8–700,000)	178 (15.7–13,027)	640 (0.8–700,000)	616 (26–182,922)
**Number of metastatic sites**				
≥ 3	61 (39.9%)	10 (35.7%)	30 (30.6%)	8 (29.6%)
**Previous Surgery**				
Surgery	37 (24.2%)	11 (39.3%)	23 (23.5%)	3 (11.1%)
**Type of comorbidities’**				
Cardiovascular	69 (45.1%)	–	–	–
Diabetes mellitus	52 (34%)			
Dyslipidemia	29 (18.9%)			
Respiratory	13 (8.5%)			
Genitourinary	15 (9.8%)			

Number (N); Eastern Cooperative Oncology Group Performace Status (ECOG PS); carbohydrate antigen 19.9 (CA 19.9).

The mean age at diagnosis was 67 years with a prevalence of the male gender (57.5%); Eastern Cooperative Oncology Group-Performance Status (ECOG-PS) was 1 in 80 (51.2%) patients and 0 in the remaining. Median baseline carbohydrate antigen (CA) 19.9 was 547 U/ml (range 0.8–700,000 U/ml). The 39.9% of the patients had more than 3 metastatic sites; 37 patients have been previously treated with surgery. Regarding comorbidities, 95 (62.3%) presented ≥ 1 comorbidity, and the most frequent (69, 45.1%) was cardiovascular; diabetes mellitus and dyslipidemia were presented in 52 (34%) and 29 (19.9%) patients, respectively, whereas respiratory and genitourinary comorbidities in the 8.5% and 9.8%, respectively.

### Population subgroups

Twenty-eight patients had a score = 0, 98 patients had a score = 1–2, and 74 a score = 3 (Table 1). In the group with score = 1–2, 28 (28.6%) patients were over 70 years old, 53 (54.1%) had ECOG-PS 1 and 68 (69.4%) presented ≥ 1 comorbidity. Male gender was represented mainly in patients with score = 3 (63%), whereas ≥ 3 metastatic sites were present in patients with score = 1. 39.3% of patients in the score = 0, 23.5% in the score = 1–2 and 11.1% in the score = 3, had previously received surgical treatment.

### Chemotherapy regimens

During the study, patients received a median of five cycles (range 1–17) of treatment; with a starting dose of nab-paclitaxel 125 mg/m^2^ plus gemcitabine 1000 mg/m^2^. Dose reduction has been necessary in 88 (57.5%) patients, 18 (64.3%) in the score = 0, 56 (57.1%) in the score = 1–2 and 14 (51.8%) in the score = 3, without significant difference between gemcitabine and nab-paclitaxel. Dose delays occurred in 51 (33.5%) patients with higher prevalence in the score = 1–2 (37.1%); treatment interruption occurred always in 51 (33.5%) patients with greater occurrence in patients with score = 0 (46.4%). GCF prophylaxis was required in 9 (33.3%) patients with score = 3 and in 4 (14.8%) and 12 (12.4%) in patients with score = 0 and score = 1–2, respectively. Over half of the patients with score = 0 received a subsequent line of therapy, while less than half in the other two groups (45.95% in score = 1–2 and 33.3% in score = 3) (Table 2).

**Table 2:** Dose reduction, treatment delay, treatment interruption and GCF- prophylaxis according to score population.

	All patients (N = 153)	Subgroup patients		
		Score = 0 (N = 28)	Score = 1–2 (N = 98)	Score = 3 (N = 27)
Cycles Median (range)	5 (1–17)	6 (1–17)	5 (1–17)	4 (1–10)
Dose reduction	88 (57.5%)	18 (64.3%)	56 (57.1%)	14 (51.8%)
Treatment delay	51 (33.5%)	8 (28.6%)	36 (37.1%)	7 (25.9%)
Treatment interruption	51 (33.5%)	13 (46.4%)	32 (32.6%)	6 (22.2%)
GCF-Prophylaxis	25 (16.4%)	4 (14.8%)	12 (12.4%)	9 (33.3%)
Subsequent line of therapy	71 (46.4%)	17 (60.7%)	45 (45.9%)	9 (33.3%)

Number (N); granulocyte-colony stimulating factor (GCF).

### Efficacy outcomes

The efficacy of chemotherapy was compared between the three groups (Table 3). Disease control rate (DCR) by Response Evaluation Criteria in Solid Tumors (RECIST) was 82.1% in score = 0 group, 61.2% in score = 1–2 group and 70.4% in patients with score = 3. Progression disease (PD) was recorded in 42 (27.4%) patients, with prevalence in score = 1–2 patients (31.6%) *vs* 22.2% and 17.9% in score = 3 and score = 0, respectively.

**Table 3:** Best response, PFS and OS according to score population.

	All patients (N = 153)	Subgroup patients		
		Score = 0 (N = 28)	Score = 1–2 (N = 98)	Score = 3 (N = 27)
PR	58 (37.1%)	16 (57.1%)	34 (34.7%)	8 (29.6%)
SD	44 (28.8%)	7 (25%)	26 (26.5%)	11 (40.7%)
DCR (PR + SD)	102 (66.7%)	23 (82.1%)	60 (61.2%)	19 (70.4%)
PD	42 (27.4%)	5 (17.9%)	31 (31.6%)	6 (22.2%)
NE	9 (5.9%)	0	7 (7.1%)	2 (7.4%)
PFS months (95% ICI) (number of events)	6 (5–6) 132	7 (5–9) 25	6 (5–7) 85	6 (4–7) 22
OS months (95% ICI) (number of events)	11 (10–13) 121	20 (12–22) 18	11 (9–13) 78	8 (6–12) 25

Number (N); partial response (PR); stable disease (SD); disease control rate (DCR); progression disease (PD); not evaluable (NE); mMedian (median); progression free survival (PFS); overall survival (OS); confidence interval (CI).

**Figure 1:**
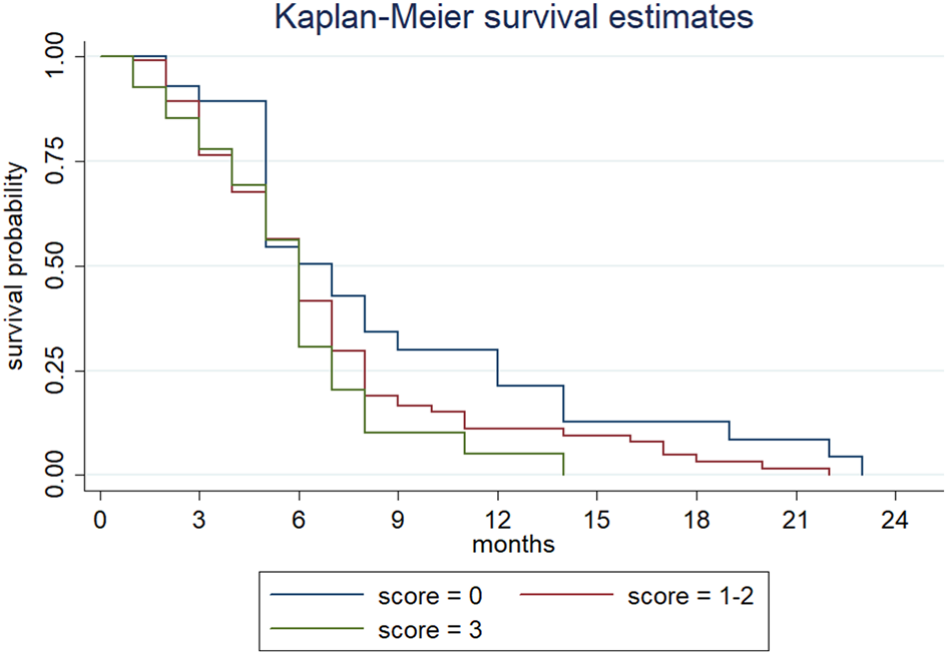
Progression free survival according to the score.

Progression free survival (PFS) was not significantly affected by age, PS, or comorbidity: 7 months in score = 0 *vs* 6 months in score = 1–2 (hazard ratio [HR] 1.18, 95% confidence interval [CI] 0.98–1.42) (*p* = 0.09) and score = 3 (HR 1.32, 95%CI 0.83–2.10) (*p* = 0.2) groups (Table 3) (Fig. 1).

**Figure 2:**
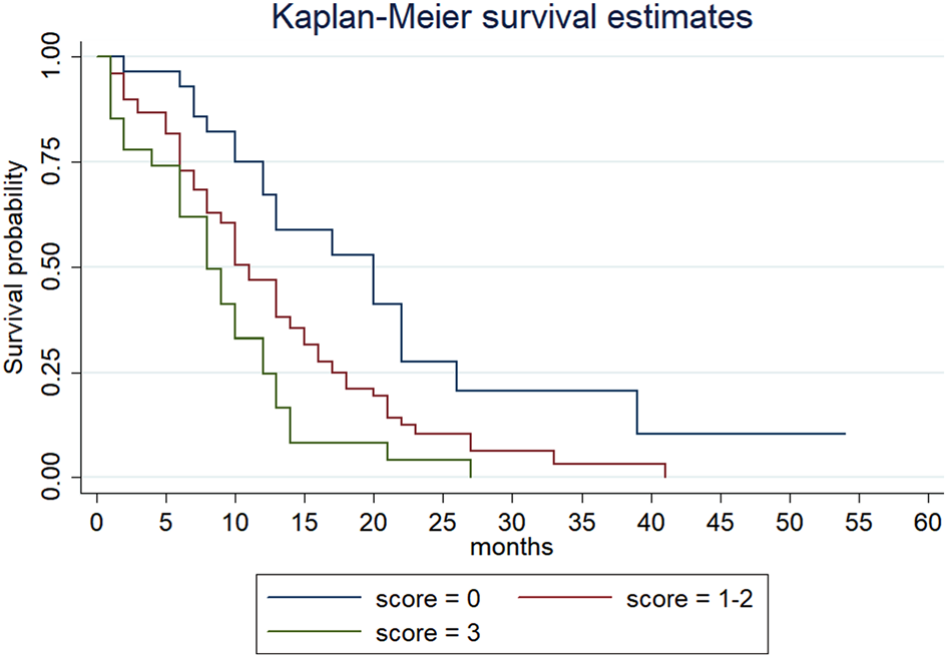
Overall survival according to the score.

Contrariwise, overall survival (OS) was significantly higher in patients with score = 0 (20 months) compared to patients with score = 1–2 (11 months) (HR 1.40, 95%CI 1.17–1.68) (*p* < 0.001) and patients with score = 3 (8 months) (HR 1.91, 95%CI 1.22–2.99) (*p* < 0.001) (Table 3) (Fig. 2).

Efficacy outcomes were estimated according to the patients score (0 or ≥ 1) and according to number of comorbidities (0 or ≥ 1). PFS was 7 and 6 months in patients with score = 0 and patients with score ≥ 1, respectively (HR 1.5, p = 0.1). OS was of 20 months in score = 0 group and 10 months in score ≥ 1 patients, with a difference statistically significant (HR 2.12, *p* < 0.001).

The difference in OS was also statistically significant in patients without comorbidities compared to patients with > 1 comorbidities (15 months *vs* 10 months) (HR 1.6, p < 0.001). Whereas PFS was the same between comorbidities groups (6 months) (HR 1.1, *p* = 0.5) (Table 1S).

Finally, a subgroup efficacy analysis according to the type of comorbidities was performed. A statistically significant difference in OS was recorded in patients with cardiovascular comorbidities (10 months) compared with patients without cardiovascular comorbidities (13 months) (HR 1.5, *p* = 0.02) (Table 2S). No differences in OS and PFS have been shown for other types of comorbidities.

## Discussion

Pancreatic cancer is primarily a disease of the elderly patients with a median age at diagnosis of 72 years. Although geriatric age is characterized by organic changes that can interfere with the oncological decision-making process, the therapeutic choice should be evaluated on the basis of the biological age of each patient defined by the performance status and the comorbidities^12^. Therefore, in elderly patients, and in the presence of comorbidities, the selection of the patient is fundamental to define the best therapeutic choice.

In elderly patients with resectable pancreatic cancer, surgical resection seems to be effective and safe^13–15^, while in patients with advanced or metastatic disease the options are palliative chemotherapy or best supportive care, but few studies discuss the better choice in this population^16^.

Literature-based evidence and guidelines support the use of single-agent therapy as the optimal treatment for elderly patients with advanced PC^17^. In a prospective observational study by Locher et al. conducted in patients aged 70 years and older a fixed-dose rate gemcitabine treatment was feasible in those with good PS and without major comorbidity^9^. Similarly, in a retrospective study by Marechal et al. gemcitabine-based regimens were effective and well tolerated in patients aged 70 years and older, although, only patients with good PS have been enrolled^10^.

However, in the last years other chemotherapy regimens, such as FOLFIRINOX (5-fluorouracil/leucovorin plus irinotecan and oxaliplatin) and NabGem have become part of the advanced/metastatic PC treatment. While the unfavorable risk-to-benefit ratio contraindicates the triplet in elderly patients, NabGem was used in several study with good efficacy and safety results^18,19^. The NAPOLEON study, examined the efficacy and safety of gemcitabine plus nab-paclitaxel in older patients with mPC, especially those ≥ 75 years old. Patients were divides in two group “older” if ≥ 75 years old and “not older” if < 75 years old. The initial dose and relative dose intensities of NabGem were significantly lower in the older group. There were no significant differences in the adverse event and antitumor response rates between the two groups. Median PFS was 5.5 months and median OS was 12.0 months in the older group, versus 6.0 and 11.1 months in the non-older group, respectively^20^. Another study analyzed patients > 65 years of age with advanced PC who received a modified of gemcitabine/nab-paclitaxel in a biweekly regimen (gemcitabine 1000 mg/m^2^ and nab-paclitaxel 125 mg/m^2^ every 2 weeks on days 1 and 15 of a 28-day cycle) to evaluate efficacy and toxicity. The median OS and PFS were 9.1 months and 4.8 months, respectively. Dose reductions of gemcitabine/nab-paclitaxel were required in 10% and 4% patients, respectively^21^.

Ibusuki et al. examined the efficacy and safety of modified NabGem for 34 older patients (≥ 75 years) with advanced PC^22^. The median OS and PFS were 15.4 months and 5.9 months, respectively. The best response was partial response (PR) in 29% (10/34), stable disease (SD) in 53% (18/34), and progression disease (PD) in 15% of patients (5/34). Early discontinuation owing to intolerable adverse events occurred in one patient, and there were no chemotherapy-related deaths. The present study demonstrated that modified NabGem showed good efficacy with acceptable toxicity and that initial dose reduction may be a good option for older patients with PC to avoid early discontinuation and to maintain dose intensities^23^. However, in addition to chronological age, comorbidity is a recurring problem in the treatment of elderly patients. Comorbidities are common in cancer patients and the prevalence increases with age. Charlson Comorbidity Index (CCI), which combines age and comorbidity, is the most used index in longitudinal studies for estimating the relative risk of death from prognostic clinical covariates^24^. Although the significant effect of comorbidity on the overall survival was observed in the cancer subtypes with generally longer expected survival time (such as prostate and breast cancers), no statistically significant correlation was found in the cancers with lower life expectancy (such as pancreatic and lung cancers). Nakai et al. showed in their multivariate analysis, that CCI and PS are prognostic factors for survival in 183 patients with advanced pancreatic cancer treated with gemcitabine-based chemotherapy, contrary to age^25^. Recently, Bagni et al. showed that CCI and other factors such as diabetes, tobacco smoking, alcohol abuse, and body mass index (BMI) had no significant prognostic effect on overall survival in PC patients that received least one cycle of adjuvant or palliative chemotherapy. They confirmed instead that advanced cancer stage and poor PS were associated with increased mortality in patients with pancreatic adenocarcinoma in accordance with previous studies^26,27^. These data, although very limited, confirmed the necessity of develop new prognostic scales for patient with PC by considering the side effects of chemotherapy.

For some reasons, including a small sample size and differences in inclusion/exclusion criteria, a direct comparison between the pivotal trial and our real-world experience, was not fully possible. However, despite the limitations, we were able to confirm that the combination regimen with NabGem has resulted effective in our population compared with the randomised controlled trial sample. Specifically, we observed 11 months median OS and 6 months median PFS with a DCR of 66.7%.

Notable, the statistically significance difference in efficacy outcome observed in patients with one or more among: age ≥ 70 years old, PS = 1 and presence of at least comorbidity (score = 1–2 and score = 3) compared to < 70 years old patients with PS = 0 and without comorbidities (score = 0). In fact, OS was significantly higher in patients with score = 0 (20 months) compared to patients with score ≥ 1 (10 months) (*p* < 0.001). Conversely, no difference was recorded in PFS between two groups: 7 months in patients with score = 0 *vs* 6 months in patients with score ≥ 1 (*p* = 0.09). Furthermore, regarding comorbidities, a significant difference in OS was observed between patients with ≥ 1 comorbidities (10 months) and without comorbidities (15 months) (*p* < 0.001). Patients with cardiovascular comorbidities seem to correlate with worse overall survival (*p *= 0.02). Thus, assessment of comorbidity is important in treating patients with advanced pancreatic cancer, and stratification by dedicate index should be considered in prospective trials, especially in trials including elderly patients.

Our study has some limitation mainly owing to its retrospective nature and the absence of a standardized comparison score. On the other hand, the large sample of patients with only metastatic disease, represent a strength of the study. Moreover, in the absence of clear established criteria, we have presented a score based on three simple criteria that can be easily and quickly evaluated, which could be used in clinical practice to predict the response to treatment with NabGem. This could be useful to evaluate patients in their complexity and not simply on individual risk factors such as age, to ensure the most effective treatment available. Indeed, as showed in our analysis also patients with ≥ 70 years and in good condition, often excluded from clinical trials, can receive treatment with NabGem with the same effectiveness as the younger ones.

## Conclusion

This study showed that the combination of nab-paclitaxel and gemcitabine has a similar efficacy profile for not pretreated patients aged ≥ 70 and < 70 years. Especially elderly patients with reduced performance status (PS = 1) and comorbidities can be treated effectively with NabGem and should not be excluded from appropriate treatment based on perceptions about their life expectancy and comorbidities. However, we observed that age, performance status and comorbidities, mainly cardiovascular comorbidities, were associated with a lower OS in patients with PC compared to patients without risk factors. Therefore, given the increasing proportion of those elderly and/or comorbid patients with PC, further investigations are warranted on different risk factors (*e.g.,* age and comorbidity) in the era of aggressive cancer treatment.

## Patients and methods

### Study design and patients

Patients diagnosed with metastatic pancreatic cancer in four Italian centers between January 2015 and December 2018 were retrospectively included. All patients were deemed eligible for NabGem therapy according to the following criteria: histologically or cytologically confirmed PC; radiographically confirmed metastatic disease; no previous chemotherapy; ECOG-PS ≤ 1; adequate hemopoietic function (absolute neutrophil count [ANC] ≥ 1.5 × 109/L, hemoglobin ≥ 10 g/dL, and platelet count ≥ 100 × 109/L), liver function (bilirubin level ≤ 1.5 mg/dL, aspartate/alanine aminotransferase concentrations ≤ 2.5 times the upper limit of normal in the absence of liver metastases or less than 5 times in case of liver metastases), and renal function (serum creatinine ≤ 1.5 mg/dL or creatinine clearance ≥ 30 mL/min). Patients with history of significant cardiac disease (*e.g.,* unstable angina, uncontrolled arrhythmias, or myocardial infarction < 3 months) were excluded^28^.Patient characteristics, including age at diagnosis, gender, performance status, comorbidities, CA, number of metastases, and previous treatment.

Patients were divided into three groups by age, ECOG-PS and comorbidities. Patients presented with 1 or 2 factors between age ≥ 70 years; ECOG-PS 1; presence of at least one comorbidity were assigned a score = 1–2; to patients who had all three risk factors a score = 3, whereas a score = 0 to patients without any risk factors. In our analysis score 0 group used as reference.

This study was approved by the Local Institutional Review Board for clinical experimentation of Tuscany (Italy)—“*area vasta centro”* section, with the number:14565_oss. Written informed consent was obtained from all patients.

### Chemotherapy

Nab-paclitaxel 125 mg/m^2^, followed by gemcitabine 1000 mg/m^2^ were administered intravenously on days 1, 8 and 15 every 4 weeks according to the pivotal trial^19^. Recombinant human GCF factor and erythropoietin were administered as needed. In the event of unacceptable toxicity, doses could be reduced up to two times per therapeutic agent (to 100 or 75 mg/m2 for nab-paclitaxel and to 800 or 600 mg/m2 for gemcitabine)^19^. A missing dose within four days of the scheduled administration were considered dose delays.

### Tumor response

Before the start of treatment, a full medical history, physical examination with assessment of ECOG-PS, complete blood count with differential, full serum chemistry profile, and cardiologic assessment (*e.g.,* electrocardiogram, echocardiogram and cardiologic visit) were performed for each patient. Blood tests were performed before each therapy cycle, while measurement of the CA 19-9 serum level was performed at baseline and every 12 weeks. Tumor response was assessed via computed tomography using RECIST version 1.1^29^. The evaluation was repeated every three months or more frequently in patients with clinically suspected progression. Efficacy has been evaluated as overall survival and progression free survival. OS was defined as a time from the diagnosis of advanced pancreatic cancer to death from any cause or the date of the last follow-up visit. PFS was defined as time from the initial assessment at the cancer centre to the date of the disease progression as reported by the clinician. Disease control rate was defined as the proportion of patients with the best overall response determined as CR, PR or SD^30^.

### Statistical analysis

OS and PFS were estimated using the Kaplan–Meier method and compared using the log-rank test. Parameters with a statistically significant log-rank test were considered independent variables and included in the multivariate Cox proportional hazard regression linear model to compare HR and 95% CI. All reported *p*-values are the result of two-sided tests; *p*-values < 0.05 were supposed to indicate statistical significance. STATA v.2012 was used for statistical analysis. Prognostic factors included age (< 70 or ≥ 70 years old), PS (0 or ≥ 1) and comorbidities (0 or ≥ 1).

### Ethical approval

All procedures performed in studies involving human participants were in accordance with the ethical standards of the institutional (Local Institutional Review Board for clinical experimentation of Tuscany (Italy)—“*area vasta centro”* section, with the number:14565_oss) and/or national research committee and with the 1964 Helsinki declaration and its later amendments or comparable ethical standards.

### Informed consent

Informed consent was obtained from all individual participants included in the study.

## Supplementary Information

Supplementary Information 1.

Supplementary Information 2.

## Data Availability

The data used to support the findings of this study are available from the corresponding author upon request.
