# Supplementary material for: The impact of age, performance status and comorbidities on nab-paclitaxel plus gemcitabine effectiveness in patients with metastatic pancreatic cancer
Source: Sci Rep. 2022 May 17;12:8244. doi: 10.1038/s41598-022-12214-4 (PMC9114343; doi:10.1038/s41598-022-12214-4)
Supplement: Supplementary file 1 — Supplementary Information 1. [file 41598_2022_12214_MOESM1_ESM.docx]

**Table 1S**. PFS and OS according to the number of comorbidities

|  | PFS(95% CI) | HR(95%CI) | p | OS (95% CI) | HR (95%CI) | p |
| --- | --- | --- | --- | --- | --- | --- |
| Patients with ≥1 comorbidities  Patients without comorbidities | 6 (5-6)  6 (5-7) | 1.1 (0.76-1.55) | 0.6 | 10 (8-12)  15 (12-17) | 1.6 (1.10-2.37) | <0.001 |
| Patients with ≥2 comorbidities  Patients with <2 comorbidities | 6 (5-6)  6 (5-7) | 1 (0.72-1.55) | 0.7 | 10 (8-13)  12 (10-13) | 1.1 (0.77-1.66) | 0.5 |
| Patients with ≥3 comorbidities  Patients with <3 comorbidities | 6 (5-6)  6 (5-7) | 1 (0.61-1.55) | 0.9 | 10 (8-14)  12 (10-13) | 1 (0.61-1.59) | 0.9 |

Progression free survival (PFS); overall survival (OS); hazard ratio (HR); p-vale (p); confidence interval (CI).
