# Supplementary material for: The impact of age, performance status and comorbidities on nab-paclitaxel plus gemcitabine effectiveness in patients with metastatic pancreatic cancer
Source: Sci Rep. 2022 May 17;12:8244. doi: 10.1038/s41598-022-12214-4 (PMC9114343; doi:10.1038/s41598-022-12214-4)
Supplement: Supplementary file 2 — Supplementary Information 2. [file 41598_2022_12214_MOESM2_ESM.docx]

**Table 2S.** Efficacy and survival according to types of comorbidities.

|  | PFS  (95%CI) | HR (95%CI) | p | OS (95%CI) | HR (95%CI) | p |
| --- | --- | --- | --- | --- | --- | --- |
| Cardiovascular (N=69)  YES  NO | 6 (5-7)  6 (5-7) | 1.1 (0.75-1.50) | 0.7 | 10 (8-11)  13 (11-15) | 1.5 (1.04-2.13) | 0.02 |
| Diabetes mellitus (N=52)  YES  NO | 6 (5-6)  6 (5-7) | 1.1 (0.80-1.64) | 0.4 | 10 (8-13)  13 (10-15) | 1.2 (0.81-1.72) | 0.3 |
| Dyslipidemia (N=29)  YES  NO | 6 (5-7)  6 (5-7) | 0.9 (0.61-1.47) | 0.8 | 13 (9-14)  11 (10-13) | 0.9 (0.56-1.39) | 0.6 |
| Respiratory (N=13)  YES  NO | 5 (3-7)  6 (5-7) | 1.6 (0.90-2.88) | 0.07 | 12 (10-13)  8 (6-13) | 1.9 (0.99-3.37) | 0.1 |
| Genitourinary (N=15)  YES  NO | 6 (4-10)  6 (5-7) | 1 (0.56-1.86) | 0.9 | 9 (4-10)  12 (10-13) | 1.4 (0.80-2.42) | 0.2 |

Progression free survival (PFS); overall survival (OS); Hazard ratio (HR); p-vale (p); confidence interval (CI).
